# Association between Parent's Metabolic Syndrome and 12- to18-Year-Old Offspring's Overweight: Results from the Korea National Health and Nutrition Examination Survey (K-NHANES) 2009–2016

**DOI:** 10.1155/2020/8737912

**Published:** 2020-10-30

**Authors:** Na Yeong Lee, Kyungdo Han, Yoonji Lee, Seulki Kim, Seonhwa Lee, Yujung Choi, Moon bae Ahn, Shin Hee Kim, Won Kyoung Cho, Kyoung Soon Cho, Min Ho Jung, Yong-Gyu Park, Byung-Kyu Suh

**Affiliations:** ^1^Department of Pediatrics, Seoul St. Mary's Hospital, College of Medicine, The Catholic University of Korea, Seoul, Republic of Korea; ^2^Department of Biostatistics, College of Medicine, The Catholic University of Korea, Seoul, Republic of Korea; ^3^Department of Pediatrics, Eunpyeong St. Mary's Hospital, College of Medicine, The Catholic University of Korea, Seoul, Republic of Korea; ^4^Department of Pediatrics, Yeouido St. Mary's Hospital, College of Medicine, The Catholic University of Korea, Seoul, Republic of Korea; ^5^Department of Pediatrics, Incheon St. Mary's Hospital, College of Medicine, The Catholic University of Korea, Seoul, Republic of Korea; ^6^Department of Pediatrics, St. Vincent's Hospital, College of Medicine, The Catholic University of Korea, Seoul, Republic of Korea; ^7^Department of Pediatrics, Bucheon St. Mary's Hospital, College of Medicine, The Catholic University of Korea, Seoul, Republic of Korea

## Abstract

**Background:**

Little information is available on the association between parents' metabolic syndrome (MetS) and adolescent offspring's obesity in Korea. The aim of our study is to determine the association between parent's metabolic syndrome and offspring's obesity.

**Methods:**

The study data were obtained from the Korean National Health and Nutrition Examination Survey conducted during 2009–2016. In the present study, 3140 adolescents aged 12 to 18 years, their paternal pairs (PP, fathers = 2244), and maternal pairs (MP, mothers = 3022) were analyzed. Of these 3140 adolescents, 2637 had normal weight {age- and sex-specific body mass index (BMI) under the 85^th^ percentile}, whereas 467 were overweight (age- and sex-specific BMI over the 85^th^ percentile).

**Results:**

Offspring's overweight and central obesity were associated with all components of the PP's metabolic risk factors, including central obesity (*p* < 0.001), systolic (*p* < 0.001) and diastolic blood pressure (*p* < 0.001), glucose intolerance (*p* < 0.001), and triglyceride (*p* < 0.002) and high-density lipoprotein levels (*p*=0.049). In addition, offspring's overweight and central obesity were also associated with the metabolic risk factors of MP, including central obesity (*p* < 0.001), systolic (*p* < 0.001) and diastolic blood pressure (*p* < 0.001), glucose intolerance (*p* < 0.001), and triglyceride levels (*p* < 0.001). In multivariate logistic regression analysis, offspring's overweight was significantly and positively associated with parental central obesity (PP, adjusted odds ratio (OR) = 1.593; 95% confidence interval (CI): 1.192–2.128; MP, adjusted OR = 2.221, 95% CI: 1.755–2.812) and parental metabolic syndrome (PP, adjusted OR = 2.032; 95% CI: 1.451–2.846; MP, adjusted OR = 2.972, 95% CI: 2.239–3.964). As the number of parental metabolic risk factors increased, offspring's risk for overweight and central obesity increased (*p* for trends < 0.001).

**Conclusion:**

Parental metabolic syndrome was associated with obesity in 12- to 18-year-old offspring in Korea.

## 1. Introduction

Worldwide trends in obesity in children and adolescents have increased over the past three decades [1]. The same trends were found in Korea, showing that childhood obesity increased from 6.8% in 1998 to 10% in 2013 [2]. A systematic analysis published at 2013 reported that 23.8% of boys and 22.6% of girls were overweight or obese in developed countries [3]. In Korea, 18.4% of boys and 17.5% of girls aged 2 to 19 years were classified as overweight (BMI > 85^th^ percentile) from 2010 to 2012, based on the 2007 Child Growth Chart of Korea [4]. It is widely accepted that overweight and obese children and adolescents are at high risk for developing hypertension, impaired fasting blood glucose, dyslipidemia, and metabolic syndrome (MetS) [5]. Some studies have shown an association between weight status and metabolic comorbidity in children [6, 7]. Therefore, identifying children at higher risk of obesity and metabolic syndrome is very important for proper intervention, such as lifestyle modification.

Parents influence their children both genetically and environmentally. Several studies have reported familial aggregations of obesity [8] and metabolic syndrome among siblings [9, 10] and between parents and offspring [11–13]. However, previous studies mainly reported the association between parents and offspring's MetS or the association between parents and offspring's obesity. Few studies have focused on the effect of parental MetS on offspring's overweight or obesity. Thus, the aim of our study was to determine the association between parental MetS and offspring's obesity and identify which metabolic factors in the parents had the most powerful effect on offspring obesity. We also examined that the number of metabolic risk factors was associated with increased risk of offspring's obesity.

## 2. Methods

### 2.1. Data

The study data were obtained from the Korea National Health and Nutrition Examination Surveys (K-NHANES) conducted from 2009 to 2016 by the Korean Ministry of Health and Welfare. K-NHANES is a cross-sectional survey based on stratified multistage probability samples of Korean households representing the noninstitutionalized civilian population. The selection process for the survey is as follows. After a survey area was selected by sample design, households under investigation were extracted considering survey area border and appropriate number of households. This is an annual surveillance system which collects information about the survey subjects' demographic, social, health, and nutritional status through the following components surveys using representative country samples: a medical examination, a health interview survey, and a nutrition survey. K-NHANES data are publicly available without charge. [14]. The Institutional Review Board of the Catholic University of Korea approved this study (VC19ZASI0295).

### 2.2. Selection of Study Populations

In the present study, 3140 adolescents (1628 males and 1512 females) aged 12–18 years, their paternal pairs (PP, fathers = 2244), and maternal pairs (MP, mothers = 3022) were analyzed. Of these 3140 adolescents, 2673 had normal weight (NW, age- and sex-specific BMI under the 85^th^ percentile), whereas 467 were overweight (OW, age- and sex-specific BMI over the 85^th^ percentile) [15].

### 2.3. Anthropometric and Laboratory Measurements

Height was measured using a stadiometer (Seca, Hamburg, Germany). Weight was measured with a balance-beam scale (GL-6000-20, CASKOREA, Korea) with the participants wearing a standard gown. Waist circumference (WC) was measured to the nearest 0.1 cm at the end of normal expiration, measuring from the narrowest point between the midline of the most lateral border of the right and left iliac crest. Fasting plasma of glucose concentrations (*n* = 2854), glycosylated hemoglobin (HbA1C) (*n* = 1619), total cholesterol (TC, *n* = 2862), triglyceride (TG, *n* = 2862), high-density lipoprotein-cholesterol (HDL-C, *n* = 2862), and low-density lipoprotein-cholesterol (LDL-C, *n* = 2862) levels in the subjects were measured enzymatically using a Hitachi 747 chemistry analyzer (Daiichi, Tokyo, Japan) after the subjects fasted a minimum of eight hours overnight. Blood pressure (BP) was calculated as the mean of three successive readings using a standard Mercury sphygmomanometer (Baumanometer). Average sleep time (ST), walking time (WT, the percentage of people who walked for 30 minutes at least five days a week), and low income (LI, income lower than the 25^th^ percentile) were also determined [14].

### 2.4. Definitions of Abnormal Metabolic Risk Factors

The definition of parental MetS was adapted from the International Diabetes Federation's (IDF): central obesity was defined as waist circumference ≥ 90 cm in men and ≥80 cm in women; HDL-C <40 mg/dL in men and <50 mg/dL in women or specific treatment for a lipid abnormality; TG ≥ 150 mg/Dl; glucose ≥100 mg/dl; systolic blood pressure (SBP) ≥130 mmHg, and diastolic blood pressure (DBP) ≥85 mmHg. Based on the IDF definition for MetS, an individual should have central obesity plus any two of the other four components [16]. We used standard cutoff values of the criteria for abnormal metabolic risk factors in offspring as follows: central obesity (waist circumference ≥ 90^th^ percentile in age- and sex-specific criteria for those younger than 16, adult criteria were used for those ≥16 years old), HDL-C<40 mg/dL or <50 mg/dL in girls older than 16 years, TG ≥ 150 mg/dL, glucose ≥100 mg/dL, SBP ≥130 mmHg, and DBP ≥ 85 mmHg [17].

### 2.5. Potential Confounders

When we collected the potential confounders, first of all, we selected the factors associated obesity and metabolic syndrome and they should be considered clinically. Those were represented by age, gender, exercise, smoking, drinking, sleep time, and diagnostic criteria of metabolic syndrome. Data regarding the duration and frequency of subjects in walking, smoking, and drinking status were collected using a questionnaire in the presence of trained personnel to answer any questions that subjects might have. Items such as smoking and drinking were classified as if the subjects had ever smoked and drunk, and exercise was defined as participating in walking activity for >30 min per day, on more than five days per week. And, we also referenced previous studies about the association of metabolic syndrome [11, 13, 18] and obesity [8] between parents and offspring. In addition, the factors from the statistically significant results of our subjects were also considered.

### 2.6. Statistical Analysis

All statistical analyses were performed using SAS Version 9.3 (SAS Institute Inc., Cary, USA). Sampling weights were incorporated to produce valid population estimates that accounted for the complex survey design of the K-NHANES. The data are presented as mean ± standard error (SE) for continuous variables and frequency percentage for categorical variables. The characteristics and laboratory data of the body composition groups by sex were compared using ANOVA for continuous measures and chi-squared tests for categorical measures. Multivariate logistic regression models were used to compare the adjusted ORs and 95% CIs of the metabolic risk factors between body composition groups after adjusting for potentially confounding variables.

## 3. Results

1. Characteristics and anthropometric data of 12- to 18-year-old adolescents and parents (Table 1).
2. In OW adolescents, weight (*p* < 0.001), BMI (*p* < 0.001) and WC (*p* < 0.001) were higher than those in NW adolescents. The ST per a day (*p*=0.006) of OW adolescents was shorter than that of NW adolescents. The mean BMI (*p* < 0.001), WC (*p* < 0.001), SBP (*p*=0.004), DBP (*p*=0.001), fasting glucose (*p*=0.002), and TG (*p*=0.007) in the PP of the OW adolescents were higher than those in the PP of NW adolescents. The mean BMI (*p* < 0.001), WC (*p* < 0.001), SBP (*p*=0.004), fasting glucose (*p* < 0.001), and TG (*p* ≤ 0.001) in the MP of OW adolescents were higher than those in the MP of NW adolescents.
3. Correlation between metabolic risk factors of 12- to 18-year-old adolescents with parents (Table 2).
4. The mean BMI of offspring was positively correlated with WC (*r* = 0.223, *p* < 0.001), SBP (*r* = 0.095, *p* < 0.001), DBP (*r* = 0.093, *p* ≤ 0.001), fasting glucose (*r* = 0.101, *p* ≤ 0.001), and TG (*r* = 0.083, *p*=0.002). However, it was negatively correlated with HDL (*r* = −0.048, *p*=0.049) of the PP. The mean BMI of the offspring was positively correlated with waist circumference (*r* = 0.235, *p* < 0.001), SBP (*r* = 0.096, *p* < 0.001), DBP (*r* = 0.067, *p* ≤ 0.001), fasting glucose (*r* = 0.140, *p* < 0.001), and TG (*r* = 0.091, *p* < 0.001) of the MP.
5.  The mean WC of the offspring was positively correlated with WC (*r* = 0.244, *p* < 0.001, SBP (*r* = 0.075, *p*=0.003), DBP (*r* = 0.054 *p*=0.029), fasting glucose (*r* = 0.101, *p* ≤ 0.001), and TG (*r* = 0.079, *p*=0.002). However, it was negatively correlated with the HDL values (*r* = -0.052, *p*=0.026) of the PP. The mean WC of the offspring was positively correlated with WC (*r* = 0.261, *p* < 0.001), SBP (*r* = 0.098, *p* < 0.001), DBP (*r* = 0.068, *p* ≤ 0.001), fasting glucose (*r* = 0.122, *p* < 0.001), and TG (*r* = 0.088, *p* < 0.001) but negatively correlated with HDL (*r* = −0.028, *p*=0.151) values of the MP.
6. Multivariate logistic regression analyses of overweight status and central obesity in 12- to 18-year-old adolescents according to metabolic risk factors of their parents (Table 3).
7. The OR of being overweight was significantly increased in adolescents with PP with metabolic risk factors, including central obesity (OR = 1.593, 95% CI: 1.192–2.128), high blood pressure (OR = 1.431, 95% CI: 1.091–1.877), impaired fasting glucose (OR = 1.711, 95% CI: 1.304–2.246), high TG (OR = 1.402, 95% CI: 1.066–1.845), and PP with MetS (OR = 1.651, 95% CI: 1.245–2.190) after adjusting for age, sex, smoking, drinking, exercise, income, and average daily ST of the offspring and smoking, drinking, exercise, income, and average daily ST of the PP.
8.  The OR of being overweight in the offspring was significantly increased in those with MP with metabolic risk factors, including central obesity (OR = 2.221, 95% CI: 1.755–2.812), high blood pressure (OR = 1.299, 95% CI: 0.992–1.701), impaired fasting glucose (OR = 1.857, 95% CI: 1.443–2.391), high TG (OR = 1.249, 95% CI: 0.928–1.681), and MP with MetS (OR = 1.706, 95% CI: 1.408–2.212) after adjusting for age, sex, smoking, drinking, exercise, income, and average daily ST of the offspring and smoking, drinking, exercise, income, and average daily ST of the MP.
9.  The OR of having central obesity in offspring was significantly increased in PP with metabolic risk factors, including central obesity (OR = 2.032, 95% CI: 1.451–2.846), high blood pressure (OR = 1.618, 95% CI: 1.185–2.210), impaired fasting glucose (OR = 1.561, 95% CI: 1.123–2.710), high TG {OR = 1.426, 95% CI: 1.027–1.981), and PP with MetS (OR = 1.935, 95% CI: 1.397–2.682) after adjusting for age, sex, smoking, drinking, exercise, income, and average daily ST of the offspring and smoking, drinking, exercise, income, and average daily ST of the PP.
10. The OR of having central obesity in offspring was significantly increased in those with MP with metabolic risk factors, including central obesity (OR = 2.979, 95% CI: 2.239–3.964), high blood pressure (OR = 1.326, 95% CI: (0.955–1.841), impaired fasting glucose (OR = 1.927, 95% CI: 1.443–2.590), high TG (OR = 1.279, 95% CI: 0.899–1.820), and MP with MetS (OR = 1.858, 95% CI: 1.361–2.538) after adjusting for age, sex, smoking, drinking, exercise, income, and average daily ST of the offspring and smoking, drinking, exercise, income, and average daily ST of the MP.
11. Multivariate logistic regression analysis of offspring with OW status and central obesity according to the number of metabolic risk factors of their parental pairs (Table 4).
12.  As the number of metabolic risk factors in the PP increased, the OR of being OW in the offspring also increased, from 1.537 for only one metabolic risk factor to 2.716 for five metabolic risk factors (*p* < 0.001 for trends) after adjusting for age, sex, drinking, smoking, exercise, income, and average daily ST of offspring and smoking, drinking, exercise, income, and average daily ST of the PP. Similarly, the number of metabolic risk factors in the MP also influenced the OR of being overweight in the offspring, with the same trends of as that of the PP. The OR increased from 1.102 for one metabolic risk factor to 5.057 for five metabolic risk factors (*p* < 0.001 for trends) after adjusting for age, sex, drinking, smoking, exercise, income, and average daily ST of offspring and smoking, drinking, exercise, income, and average daily ST of the MP.
13.  The OR of central obesity in the offspring was also influenced by the number of parental metabolic risk factors. As the number of metabolic risk factors in the PP increased from 1 to 5, the OR of being central obese in the offspring increased from 1.019 to 2.528 (*p* < 0.001 for trends) after adjusting for age, sex, drinking, smoking, exercise, income, and average daily ST of offspring and smoking, drinking, exercise, income, and average daily ST of the PP. The number of metabolic risk factors in the MP also influenced the OR of being central obese in the offspring with the same trends. The OR of being central obese in the offspring increased from 1.439 to 6.512 (*p* < 0.001 for trends) when the number of metabolic risk factor increased from 1 to 5 after adjusting for age, sex, drinking, smoking, exercise, income, and average daily ST of offspring and smoking, drinking, exercise, income, and average daily ST of the MP.

## 4. Discussion

In this study, we found that the risk of being overweight and central obese in the offspring was considerably associated with parental MetS. In fathers, all five components of MetS were associated with offspring's BMI and WC. All mother's metabolic risk factors except HDL were also associated with offspring's obesity. Among parental metabolic risk factors, WC of both PP and MP had the highest correlation coefficient with offspring's BMI and WC. The OR of being OW and having central obesity were increased in the offspring with paternal or maternal MetS, respectively. As the number of parental metabolic risk factors increased, the odds ratio of being OW and central obese was also increased.

Several studies reported associations of OW and obesity in offspring [8] with metabolic syndrome [11–13, 19] in parents, although such associations are influenced by sex and age. In our study, we found that the parents of OW adolescents had higher BMIs and WCs than the parents of NW adolescents, which could indicate a familial aggregation of obesity. And, we also found that parental MetS was associated with metabolic risk factors in the offspring. Existing studies have mainly focused on the association of obesity or MetS between parents and offspring. However, we analyzed the parents' individual MetS factors and confirmed that each factor could affect OW status and central obesity in their offspring. Thus, attention should be paid to children if their parents have metabolic risk factors, even if there is only one risk factor, instead of merely focusing on the children of parents diagnosed with MetS.

Yoo el al. [11] and Lee [12] reported that children had a higher prevalence of MetS if their parents had MetS in South Korea. Regardless of nationality, race, and region, we could find the similar results of studies about the association between parents and offspring in MetS [10, 13, 19, 20]. However, few studies have paid attention to the fact that as the number of parental MetS factor increases, the risk of OW and central obesity also increased in their children. The results of the present study demonstrated a graded effect of the number of parental metabolic risk factors on their adolescent offspring. This suggests the importance of reducing at least one metabolic risk factor in parents to decrease the risk of OW or obese children.

In addition, another important finding in our study that was not noticeable in other studies was the difference of the influence of mother and father to offspring's obesity. As mentioned before, it showed same trend that as the number of parental metabolic risk factors increases, and offspring's risk of being obese also increased in both parents. But, there is a distinct difference in the degree of risk that each mother and father have on their child's obesity; the number of MP's metabolic risk factors were more potent risk factors to offspring's obesity.

It is well known that MetS pathophysiology can be explained by glucose metabolic disorders, dyslipidemia, obesity, and hypertension [21]. Central obesity could be an independent predictor of insulin resistance, lipid levels, and blood pressure [22, 23]. Some studies have reported WC might reflect adiposity in children [24]. From this study, we learn that parental MetS, especially, central obesity was a noteworthy factor that might affect weight and central obesity in their offspring. Thus, it is important to check WC, in addition to weight, for monitoring central obesity and preventing progression to diseases associated with obesity, such as cardiovascular disease and type 2 diabetes mellitus (DM).

Children with obesity are more likely to have obesity-related morbidity [25]. Being overweight and having central obesity are not static health problems. They could lead to metabolic syndrome, cardiovascular diseases, and type 2 DM [26] later in life [27–29]. Therefore, it is very important to manage obesity in children and adolescents. The prevention and management of offspring obesity should pay attention to factors identified in this study. From this study, we know which parental metabolic risk factors had the biggest effect on offspring obesity. Thus, we should assess parental metabolic risk and identify children with the potential for developing obesity, so proper interventions, such as diet, exercise, and lifestyle modification, can be applied.

Our study had several limitations. First, this study was a cross-sectional study. We could not follow-up to determine the causality of obesity. Second, this study did not compare the association between spousal or sibling conditions which can help analysis of familial aggregation. However, after correcting factors related parent's and offspring's behavior, the relationship between parent's metabolic syndrome and offspring's obesity. Therefore, it is assumed that biological mechanism is stronger than environmental effect, but more detailed research is needed in near future. Third, we could not analyze diet as confounder although we recognized that diet could be one of the causes of metabolic syndrome. That is because it did not perform the same type of question about diet during the study periods. We think further research reflects diet as confounder is needed sooner or later. However, in this study, we reported the association between parental MetS and offspring obesity, and parental central obesity was the most powerful single factor affecting offspring obesity. These results suggest that the prevention and management of offspring obesity should consider various aspects, including a powerful parental effect on their children's health.

## Figures and Tables

**Table 1:** Characteristics and laboratory data according to obesity in 12- to 18-year-olds Korean adolescents and their parents.

	Normal	Overweight	*p*-value
Number	2673	467	
Male	53.0 (1.1)	51.8 (2.6)	0.6656
Age	15.2 ± 0.04	15.3 ± 0.1	0.7011
Height	165.2 ± 0.1	166.0 ± 0.4	0.1369
Weight	55.2 ± 0.2	76.6 ± 0.7	<0.0001
BMI	20.1 ± 0.06	27.6 ± 0.1	<0.0001
WC	68.7 ± 0.1	85.9 ± 0.4	<0.0001
Average sleep time	7.1 ± 0.03	6.9 ± 0.07	0.0063
Smoking (lifelong smoking)	18.0 (0.8)	18.6 (2.1)	0.8013
Drinking (lifelong drinking)	39.8 (1.1)	41.1 (2.6)	0.6488
Walking time	50.5 (1.1)	52.2 (2.7)	0.5562
Low income	9.9 (0.7)	13.6 (2.0)	0.0543

Father	1915	329	
Age	46.8 ± 0.1	46.6 ± 0.3	0.5918
BMI	24.2 ± 0.08	25.9 ± 0.1	<0.0001
WC	84.7 ± 0.2	88.4 ± 0.5	<0.0001
SBP	118.4 ± 0.3	121.2 ± 0.8	0.0042
DBP	80.7 ± 0.2	82.9 ± 0.6	0.0015
GLU	99.8 ± 0.6	104.4 ± 1.4	0.0029
TG∗	131.2 (127.7–134.8)	145.0 (135.6–155.0)	0.0073
HDL	46.8 ± 0.2	45.8 ± 0.6	0.1809
LDL	116.4 ± 0.8	114.9 ± 2.0	0.5067
TC	193.1 ± 0.8	193.7 ± 2.2	0.7916
Average sleep time	6.7 ± 0.04	6.5 ± 0.08	0.0064
Spouse	96.1 (0.5)	95.1 (1.2)	0.6195
Smoking	43.3 (1.3)	52.0 (3.2)	0.0116
Drinking	24.0 (1.1)	31.9 (2.9)	0.0067
Walking time	32.6 (1.2)	32.2 (2.9)	0.2540

Mother	2572	450	
Age	43.8 ± 0.1	43.6 ± 0.2	0.4604
BMI	23.0 ± 0.08	24.7 ± 0.2	<0.0001
WC	76.6 ± 0.2	80.7 ± 0.5	<0.0001
SBP	111.1 ± 0.3	113.4 ± 0.7	0.0047
DBP	73.7 ± 0.2	74.7 ± 0.5	0.0695
GLU	94.1 ± 0.3	99.6 ± 1.2	<0.0001
TG	88.4 (86.3–90.6)	99.1 (94.0–104.5)	0.0002
HDL	53.6 ± 0.2	53.6 ± 0.6	0.9145
LDL	112.3 ± 0.6	113.3 ± 1.5	0.5753
TC	186.3 ± 0.7	189.7 ± 1.6	0.0652
Average sleep time	6.7 ± 0.03	6.5 ± 0.07	0.0038
Spouse	90.0 (0.7)	87.6 (1.9)	0.2101
Smoking	5.4 (0.5)	8.2 (1.5)	0.0519
Drinking	4.9 (0.5)	10.3 (1.6)	00.0001
Walking time	37.9 (1.1)	41.3 (2.6)	0.2346

Abbreviations: BMI, body mass index; WC, waist circumference; SBP, systolic blood pressure; DBP, diastolic blood pressure; GLU, glucose; TG, triglyceride; HDL, high-density lipoprotein; LDL, low-density lipoprotein; TC, total cholesterol; Average sleep time, average sleep time per day; walking time, percentage of people who walked for 30 minutes at least five days a week; low income, income lower than the 25^th^ percentile; drink, average amount of alcohol consumed is 7 or more for men and 5 or more for women at least 2 days a week.

**Table 2:** Correlations of parental metabolic risk factors with BMI and WC in 12- to 18-year-old adolescents.

	BMI of offspring		WC of offspring	
	*r*	*p*	*r*	*p*
Father				
WC	0.2235	<0.0001	0.2442	<0.0001
SBP	0.0958	<0.0001	0.075	0.0037
DBP	0.0937	00.0001	0.0548	0.0296
GLU	0.1010	0.0003	0.1017	0.0002
TG	0.0834	0.0023	0.0791	0.0029
HDL	−0.0487	0.0498	−0.0528	0.0263

Mother				
WC	0.2355	<0.0001	0.2615	<0.0001
SBP	0.0968	<0.0001	0.0987	<0.0001
DBP	0.0678	0.0006	0.0689	0.0005
GLU	0.1400	<0.0001	0.1228	<0.0001
TG	0.0918	<0.0001	0.0886	<0.0001
HDL	−0.0358	0.0818	−0.0388	0.0574

Abbreviations: BMI, body mass index; WC, waist circumference; SBP, systolic blood pressure; DBP, diastolic blood pressure; GLU, glucose; TG, triglyceride; HDL, high-density lipoprotein.

**Table 3:** Multivariate logistic regression analyses of overweight status and central obesity in offspring with parental metabolic risk factors.

	Or (95% CI)	
	Overweight of offspring	Central obesity of offspring
Father		
WC	1.913 (1.557,2.350)	2.424 (1.823,3.223)
HDL	1.015 (0.817,1.263)	1.080 (0.801,1.458)
BP	1.511 (1.244,1.836)	1.551 (1.192,2.019)
GLU	1.458 (1.200,1.772)	1.427 (1.077,1.890)
TG	1.314 (1.078,1.602)	1.437 (1.081,1.912)
MetS	1.650 (1.352,2.013)	2.014 (1.535,2.644)

Mother		
WC	1.881 (1.572,2.250)	2.661 (2.066,3.429)
HDLS	1.035 (0.860,1.244)	1.086 (0.838,1.407)
BP	1.324 (1.075,1.630)	1.338 (1.008,1.775)
GLU	1.715 (1.411,2.084)	1.838 (1.418,2.383)
TG	1.407 (1.134,1.746)	1.405 (1.042,1.892)
MetS	1.715 (1.408,2.089)	1.834 (1.398,2.405)

Abbreviations: OR, odds ratio; CI, confidence interval; BMI, body mass index; WC, waist circumference; HDL, high-density lipoprotein; BP, blood pressure; GLU, glucose; TG, triglyceride; MetS, metabolic syndrome. Adjusted for age, sex, smoking, drinking, exercise, income and average sleep time per a day of offspring, and smoking, drinking, exercise, income, and average sleep time per a day of parents.

**Table 4:** Multivariate logistic regression analyses of overweight status and central obesity offspring according to the number of metabolic risk factors of the parents.

	Number of MetS	Overweight of offspring			Central obesity of offspring		
		OR	Lower	Upper	OR	Lower	Upper
Father	0	1			1		
	1	1.537	0.937	2.52	1.019	0.563	1.843
	2	2.138	1.335	3.423	1.784	1.03	3.089
	3	2.594	1.586	4.244	2.396	1.363	4.212
	4	2.648	1.527	4.591	2.625	1.400	4.921
	5	2.716	1.311	5.626	2.528	1.021	6.261
	*p* for trend	<0.0001			<0.0001		

Mother	0	1			1		
	1	1.102	0.805	1.511	1.439	0.968	2.141
	2	1.995	1.407	2.830	2.348	1.533	3.594
	3	1.974	1.376	2.831	2.543	1.602	4.037
	4	2.008	1.275	3.161	2.411	1.388	4.190
	5	5.057	2.278	11.22	6.512	2.737	15.49
	*p* for trend	<0.0001			<0.0001		

Abbreviations: BMI, body mass index; WC, waist circumference; OR, odds ratio. Adjusted for age, sex, smoking, drinking, exercise, income, and average sleep time per day of offspring, and smoking, drinking, exercise, income, and average sleep time per day of parents.

## Data Availability

The data used to support this study are available at https://knhanes.cdc.go.kr/knhanes/sub04/sub04_03.do?classType=7 (Korea Centers for Disease Control and Prevention and Korean National Health and Nutrition Examination Survey)

## References

[1] Wang Y., Lobstein T. (2006). Worldwide trends in childhood overweight and obesity. *International Journal of Pediatric Obesity*.

[2] Ha K. H., Kim D. J. (2016). Epidemiology of childhood obesity in Korea. *Endocrinology and Metabolism*.

[3] Ng M., Fleming T., Robinson M. (2014). Global, regional, and national prevalence of overweight and obesity in children and adults during 1980–2013: a systematic analysis for the Global Burden of Disease Study 2013. *The Lancet*.

[4] Bahk J., Khang Y.-H. (2016). Trends in measures of childhood obesity in Korea from 1998 to 2012. *Journal of Epidemiology*.

[5] Bradford N. F. (2009). Overweight and obesity in children and adolescents. *Primary Care: Clinics in Office Practice*.

[6] Lim H., Xue H., Wang Y. (2014). Association between obesity and metabolic co-morbidities among children and adolescents in South Korea based on national data. *BMC Public Health*.

[7] Cho W. K., Han K., Ahn M. B. (2018). Metabolic risk factors in Korean adolescents with severe obesity: results from the Korea national health and nutrition examination surveys (K-nhanes) 2007-2014. *Diabetes Research and Clinical Practice*.

[8] Naess M., Holmen T. L., Langaas M., Bjorngaard J. H., Kvaloy K. (2016). Intergenerational transmission of overweight and obesity from parents to their adolescent offspring - the HUNT study. *PloS One*.

[9] Sung J., Lee K., Song Y.-M. (2009). Heritabilities of the metabolic syndrome phenotypes and related factors in Korean twins. *The Journal of Clinical Endocrinology & Metabolism*.

[10] Feng Y., Zang T., Xu X., Xu X. (2008). Familial aggregation of metabolic syndrome and its components in a large Chinese population. *Obesity*.

[11] Yoo E.-G., Park S. S., Oh S. W., Nam G.-B., Park M. J. (2012). Strong parent-offspring association of metabolic syndrome in Korean families. *Diabetes Care*.

[12] Lee K. (2017). Metabolic syndrome in Korean adolescents and young adult offspring and their parents. *Asia Pacific Journal of Clinical Nutrition*.

[13] Khan R. J., Gebreab S. Y., Riestra P., Xu R., Davis S. K. (2014). Parent-offspring association of metabolic syndrome in the framingham heart study. *Diabetology & Metabolic Syndrome*.

[14] Korea centers for disease control and prevention (2016). Korean national health and nutrition examination survey. https://knhanes.cdc.go.kr/knhanes/sub04/sub04_03.do?classType=7.

[15] Kim J. H., Yun S., Hwang S.-s. (2018). The 2017 Korean national growth charts for children and adolescents: development, improvement, and prospects. *Korean Journal of Pediatrics*.

[16] Alberti K. G. M., Zimmet P., Shaw J. (2005). The metabolic syndrome-a new worldwide definition. *The Lancet*.

[17] Wittcopp C., Conroy R. (2016). Metabolic syndrome in children and adolescents. *Pediatrics in Review*.

[18] Park H. S., Park J. Y., Cho S.-I. (2006). Familial aggregation of the metabolic syndrome in Korean families with adolescents. *Atherosclerosis*.

[19] Sabo R. T., Lu Z., Deng X. (2012). Parental and offspring associations of the metabolic syndrome in the fels longitudinal study. *The American Journal of Clinical Nutrition*.

[20] Baxi R., Vasan S. K., Hansdak S. (2016). Parental determinants of metabolic syndrome among adolescent Asian Indians: a cross-sectional analysis of parent-offspring trios. *Journal of Diabetes*.

[21] Gluvic Z., Zaric B., Resanovic I. (2017). Link between metabolic syndrome and insulin resistance. *Current Vascular Pharmacology*.

[22] Bacha F., Saad R., Gungor N., Arslanian S. A. (2006). Are obesity-related metabolic risk factors modulated by the degree of insulin resistance in adolescents?. *Diabetes Care*.

[23] Lee S., Bacha F., Arslanian S. A. (2006). Waist circumference, blood pressure, and lipid components of the metabolic syndrome. *The Journal of Pediatrics*.

[24] Maynard L. M., Wisemandle W., Roche A. F., Chumlea W. C., Guo S. S., Siervogel R. M. (2001). Childhood body composition in relation to body mass index. *Pediatrics*.

[25] Barlow S. E. (2007). Expert committee recommendations regarding the prevention, assessment, and treatment of child and adolescent overweight and obesity: summary report. *Pediatrics*.

[26] Reis E. C., Kip K. E., Marroquin O. C. (2006). Screening children to identify families at increased risk for cardiovascular disease. *Pediatrics*.

[27] Chien K.-L., Hsu H.-C., Sung F.-C., Su T.-C., Chen M.-F., Lee Y.-T. (2007). Metabolic syndrome as a risk factor for coronary heart disease and stroke: an 11-year prospective cohort in Taiwan community. *Atherosclerosis*.

[28] Wilson P. W. F., D’Agostino R. B., Parise H., Sullivan L., Meigs J. B. (2005). Metabolic syndrome as a precursor of cardiovascular disease and type 2 diabetes mellitus. *Circulation*.

[29] Hwang Y.-C., Jee J.-H., Oh E. Y. (2009). Metabolic syndrome as a predictor of cardiovascular diseases and type 2 diabetes in Koreans. *International Journal of Cardiology*.

